# Supplementary figures and images for: Non-SUMOylated CRMP2 decreases NaV1.7 currents via the endocytic proteins Numb, Nedd4-2 and Eps15
Source: Mol Brain. 2021 Jan 21;14:20. doi: 10.1186/s13041-020-00714-1 (PMC7819318; doi:10.1186/s13041-020-00714-1)

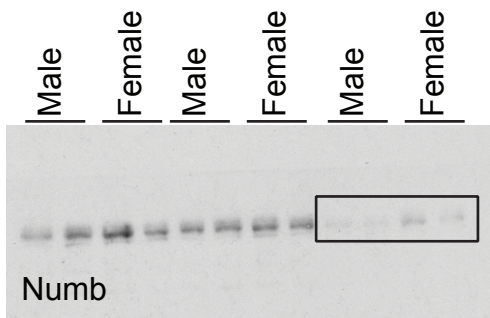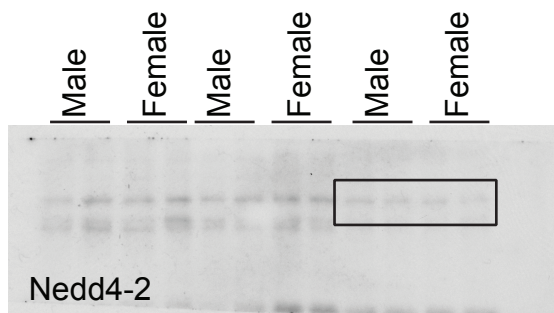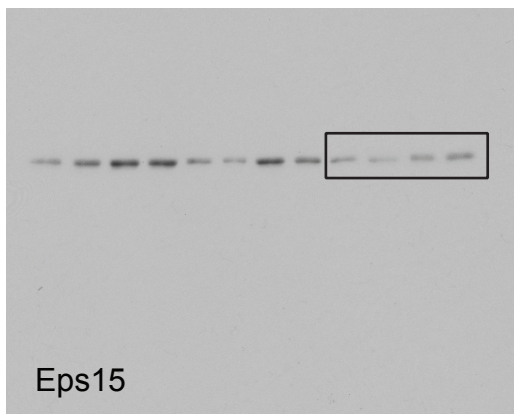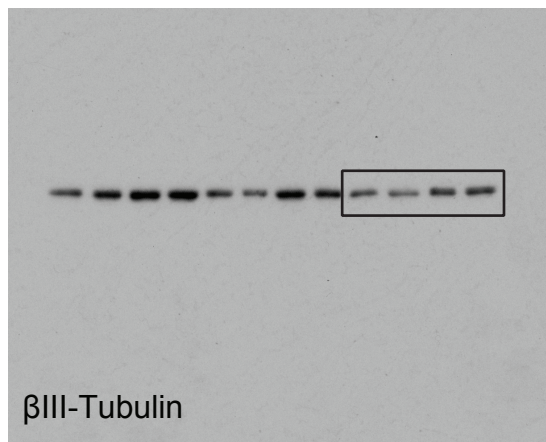

Supplement: Supplementary file 2 — Additional file 2: Figure S1. Raw immunoblot of Numb, Nedd4-2 and Eps15 expression in male and female mice DRGs. Raw Western blots showing the expression of the endocytic proteins Numb, Nedd4-2 and Eps15 in lumbar DRG from male and female wildtype mice. βIII-tubulin served as a loading control. [file 13041_2020_714_MOESM2_ESM.pdf]
